# An Intelligent Ensemble Neural Network Model for Wind Speed Prediction in Renewable Energy Systems

**DOI:** 10.1155/2016/9293529

**Published:** 2016-03-01

**Authors:** V. Ranganayaki, S. N. Deepa

**Affiliations:** Department of Electrical and Electronics Engineering, Anna University, Regional Campus Coimbatore, Coimbatore, Tamil Nadu 641 046, India

## Abstract

Various criteria are proposed to select the number of hidden neurons in artificial neural network (ANN) models and based on the criterion evolved an intelligent ensemble neural network model is proposed to predict wind speed in renewable energy applications. The intelligent ensemble neural model based wind speed forecasting is designed by averaging the forecasted values from multiple neural network models which includes multilayer perceptron (MLP), multilayer adaptive linear neuron (Madaline), back propagation neural network (BPN), and probabilistic neural network (PNN) so as to obtain better accuracy in wind speed prediction with minimum error. The random selection of hidden neurons numbers in artificial neural network results in overfitting or underfitting problem. This paper aims to avoid the occurrence of overfitting and underfitting problems. The selection of number of hidden neurons is done in this paper employing 102 criteria; these evolved criteria are verified by the computed various error values. The proposed criteria for fixing hidden neurons are validated employing the convergence theorem. The proposed intelligent ensemble neural model is applied for wind speed prediction application considering the real time wind data collected from the nearby locations. The obtained simulation results substantiate that the proposed ensemble model reduces the error value to minimum and enhances the accuracy. The computed results prove the effectiveness of the proposed ensemble neural network (ENN) model with respect to the considered error factors in comparison with that of the earlier models available in the literature.

## 1. Introduction

For the past decade, the energy crisis is a major problem in various countries and the renewable energy utilization is becoming important all over the world. For the growing economy, the source of energy plays a major role and deriving the energy from the wind resources, which are available in plenty, will lead to developing an energy model and aid in appropriate allocation of the resources. Wind is a natural resource and form of renewable energy available in large. Wind energy is observed to be a clean energy and also it is pollution-free. Basically, wind is characterized by its direction, speed, and the time at which it occurs. Deriving wind energy from the natural wind flow is based on the force in which it moves or actually the speed of the wind. The force of wind or the wind speed is generally nonlinear and fluctuating in nature. In spite of its original nature, wind possesses the capability to generate the required amount of energy for the regular demands of the country. The prediction of wind speed is to be carried out for enhancing the energy generated [1]. Wind speed prediction compromises between the required demand and the generated energy. Wind speed predicted model designed with high accuracy and reliability acts as an effective tool to optimize the operating cost and improve the operational feature of the grid system.

This research paper contributes development of neural network (NN) models for performing effective wind speed prediction. Predicting wind speed is an important measure for wind energy connected grid systems. Few other factors that affect wind speed include humidity, moisture in the atmospheric air, atmospheric pressure, temperature, and rainfall. Predicting accurate wind speed enables the energy people to plan accordingly for the required energy demands. Various applications of wind speed prediction are energy to the grid, satellite and rocket launch, energy for agriculture, control module operations of military sectors, and so on. This prediction protects the generation of secured wind power and enables integrating the wind energy into electricity grids.

Over the years, it has been well noted that artificial neural network models are employed for various prediction applications [2]. Artificial neural network is a computational intelligent technique resembling the characteristic of the human biological neural network. The main characteristics of neural network include nonlinearity, adaptability, ability to handle large data, and nature of generalization. Due to these inbuilt features, neural network proves itself to be an effective tool for the accurate prediction of wind speed based on the defined input parameters. Neural networks have been applied in numerous fields like prediction, recognition, image processing, classification, association, control, and so on. Several approaches [3–5] are used to increase the accuracy of wind speed prediction, including physical and statistical methods. The physical method uses simple and higher order equations and involves physical quantities of the real time system. The statistical approaches carry out the relation between the existing and forecasted output whose parameters are estimated with the available data [6]. The statistical methods work on both linear and nonlinear models.

Generally, the wind speed is observed to vary rapidly due to its nonlinear nature. ANN being a flexible and an effective tool is employed in this research paper for predicting nonlinear behavior of the considered wind prediction system. The neural networks are basically inspired from the biological functioning of the human brain model comprising their fundamental element as the artificial neuron [7]. ANN does not require mathematical equations or mathematical model of the system but tends to minimize the error automatically based on the available knowledge of inputs and outputs. Thus, this paper mainly focused on wind speed prediction using neural network models. The performance metric considered is the mean square error value employed for measuring the quality of forecasted wind speed using the neural network. When the training process is initiated, the generalization performance is noted to differ with respect to time. Further, one of the major issues in the design of ANN is fixation of hidden neurons in the hidden layer. The presence of hidden layer and hidden neurons plays a major role for computing minimal error in artificial neural network modeling process of wind speed forecasting in renewable energy systems.

In this paper, the developed ensemble neural network model is tested with the proposed 102 criteria to fix the appropriate number of hidden neurons in the hidden layer of each MLP, Madaline, BPN, and PNN. The criteria are selected based on their satisfaction on the convergence theorem. The proposed ensemble neural network model is adapted in this research paper for application of wind speed forecasting. The main focus is to achieve the minimal error, improve the network stability, and better accuracy compared to other existing approaches [8] in order to assist planning, integration, and control of power system and wind farm.

## 2. Related Work

The key objective of this research paper is to develop certain ensemble NN models and design the number of hidden neurons to be placed in hidden layer of the considered neuronal models and apply the developed model for accurate wind speed prediction. This contribution is evolved based on the detailed analysis carried out in earlier works related to this field and is presented in this section.

Numerous methods are reported in the literature for wind speed prediction such as physical approaches, time series, statistical methods, and machine learning approaches as well. A method for wind power generation employing BPN was developed in an effort to minimize the error and yield more accurate prediction [9]. Wind speed forecasting using a Recurrent Neural Network (RNN) model was modeled with an average prediction error below 10% [10]. An advanced online software platform for wind speed prediction has been modeled by Giebel [11]. A wind speed prediction up to 1 hour, 24 hours, and 48 hours based on MLP and Elman network has been proposed by Jayaraj et al. [12]. BPA (Back Propagation Algorithm) model has been developed for predicting wind speed twenty minutes in advance [13]. Silva et al. [14] presented a model employing Radial Basis Function Neural (RBFN) network for wind speed prediction and it has been found that it performs better than MLP. Zhang and Li [15] proposed ANN in hybridization with Field Programmable Gate Array (FPGA) network using state machine. Barbounis et al. [16] developed a long-term wind speed prediction with 72 hours ahead using RNN.

The MLP network for predicting wind speed at Zaragoza employed two models: time expert and spatial expert [17]. Wind speed prediction models using Taboo Search (TS) algorithm, recurrent fuzzy neural network, and adaptive neurofuzzy inference system have also been developed to enhance the accuracy of the estimated wind speed and to reduce the computation time [18–20]. Chen et al. [21] modeled a prediction system employing OLS (Orthogonal Least Squares) algorithm which measures the hidden nodes based on RBFN. This model predicted average hourly wind speed in one hour ahead. Wu et al. [22] developed a wind speed prediction from the previous values of the same variable using BPN. Monfared et al. [23] implemented a fuzzy based NN model for wind speed forecasting. This method provides a fuzzy associative memory table based on fuzzy logic and employs fast learning process for the neural network. Soman et al. [24] proposed three different models such as Adaptive Linear Network (Adaline), BPN, and hybrid network for prediction application. Han et al. [25] proposed a wind speed prediction method based on the improved neural network in which the wind direction factor was added as input vector by analyzing the relationship between wind direction and wind speed changes.

Bhaskar et al. [26] reviewed the present status of wind speed prediction which introduces the latest model of ANN, Support Vector Machine (SVM), and Particle Swarm Optimization (PSO). The work enabled the wind farm owners to understand the current wind prediction model capabilities. Fesharaki et al. [27] proposed a model employing adaptive weighted PSO with ANN for wind speed prediction. The wind speed prediction using genetic NN based on rough set theory was introduced by Guo et al. [28]. A multiple architecture system for wind speed prediction based on MLP and RBFN has also been developed [29]. Terzi et al. [30] proposed a new hybrid model which uses ANFIS and BPN for wind speed prediction. Sajedi et al. [31] proposed a model which uses ANFIS for short-term wind speed prediction. A hybrid model is developed employing RBFN and persistence method for wind speed forecasting. The RBFN model with Grubbs test is developed by Wu et al. [32]. Shi et al. [33] proposed a hybrid forecasting model which consists of ARIMA-NN and ARIMA-SVM. Cao et al. [34] proposed a model based on RNN with five different heights of wind mill. Xinrong et al. [35] modeled a Relevance Vector Machine (RVM) and Empirical Mode Decomposition (EMD) based wind speed forecasting model. Hu et al. [36] proposed a pattern based approach for short-term wind prediction to do better than the clustering based approach. Zhang et al. [37] carried out work on a hybrid wind speed forecasting based on intelligent optimized algorithm. Based on the review made with regard to the different neural network models employed for wind speed prediction, the identified limitations include the following:

- Certain approaches developed for predicting wind speed employed random number of hidden neurons in the hidden layer; this resulted in either overfitting or underfitting problem.
- Error has not been significantly decreased in the earlier methods.
- Existing methodologies employ trial procedures and in certain cases numbers of hidden neurons are not fixed.

Thus this paper focuses on developing ensemble neural network model combining the features of MLP, BPN, Madaline, and PNN to predict the wind speed for the collected real time dataset samples. Criteria are evolved satisfying the convergence theorem to fix the accurate number of hidden neurons for each of the ensembles and the average error is reduced to a possible minimal level during the training process of the developed ensemble neural network model.

## 3. Problem Formulation

The wind power generated by a wind farm is critically based on the stochastic nature of wind speed and an unexpected deviation in the wind power output results in increase in the operating costs of the electrical system under consideration. The relation between wind speed and wind power is highly nonlinear in nature. Thus presence of error in wind speed prediction will also generate a large error in wind power generation. This technique practically improves the rate of performance. Generally, the neural network learns from the past data and with that experience predicts the future data. An accurate wind speed prediction model will allow grid operations to operate economically to meet the demands of the needful electrical customers. Hence accurate and reliable wind speed prediction is a prerequisite for good grid operation and advanced control strategy. The behavior of the wind speed is nonstationary and this indicates dynamic property during different period resulting in variations of input and output [38–41].

Considering the above facts, the problem to be addressed in this paper includes formulating suitable criterion to fix the number of hidden neurons for the proposed ensemble neuronal model so as to predict wind speed with higher accuracy rate and minimal error. No proper fixation of hidden neurons for the neural network models may increase the computational time and may delay the convergence of the network increasing the error rate. Thus hidden neuron selection plays a major role in neural network modeling process and this is carried out on evaluation of the generalization error during the learning process. When a smaller number of hidden neurons are placed in the model, this may result in local minima problem and when a bigger number of hidden neurons are placed, this may lead to instability of the neuronal model. Thus a trade-off should be maintained in proposing the criterion to fix the number of hidden neurons in the hidden layer of the proposed ensemble neuronal model.

The performance of the proposed ensemble neural network model is determined by the minimal mean square error (MSE). So the mean square error is used as the performance metric for performing the learning process for wind speed prediction. To determine the optimal ensemble NN model, MSE criteria are employed and are defined by the following equation:

(1) $$\begin{array}{c} MSE=\overset{N}{\underset{i=1}{\sum }}\frac{{\left(Y_{predict}-Y_{actual}\right)}^{2}}{N}, \end{array}$$

where *Y*_predict_ specifies the predicted output, *Y*_actual_ indicates the actual output, and *N* denotes the number of samples. The perfect design of ensemble neural network architecture is highly important for the challenge of better accuracy in predictive models.

## 4. Modeling the Proposed Ensemble Neural Network Architecture

**Figure 1:**
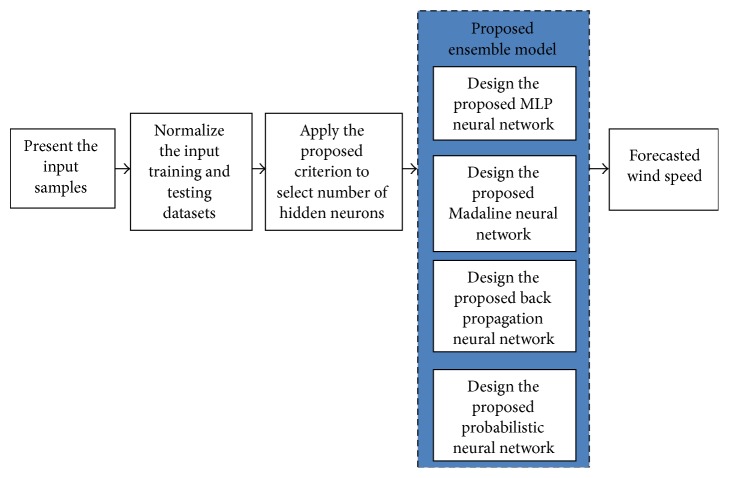
Basic block diagram of the proposed ensemble neural network model.

This section presents the proposed modeling of ensemble neural networks to be employed for wind speed prediction in renewable energy systems. Basically, in neural network modeling there exist no specific methodologies to select the number of hidden neurons to be located in the hidden layer. In this paper, certain new criteria are evolved to fix the hidden neurons for the ensemble network using the mathematical foundation of convergence theorem. Each and every criterion that satisfies the convergence theorem is tested for its optimality at the training process for reduction of errors. The criterion that achieves minimal error satisfying the convergence theorem is chosen to be the optimal criterion to be considered for fixing hidden neurons in the proposed ensemble model. The criteria are formulated based on the number of input layer neurons (*n*). The basic block diagram of the proposed ensemble neural network is as shown in Figure 1.

### 4.1. Design of the Proposed Ensemble Neural Network

**Figure 2:**
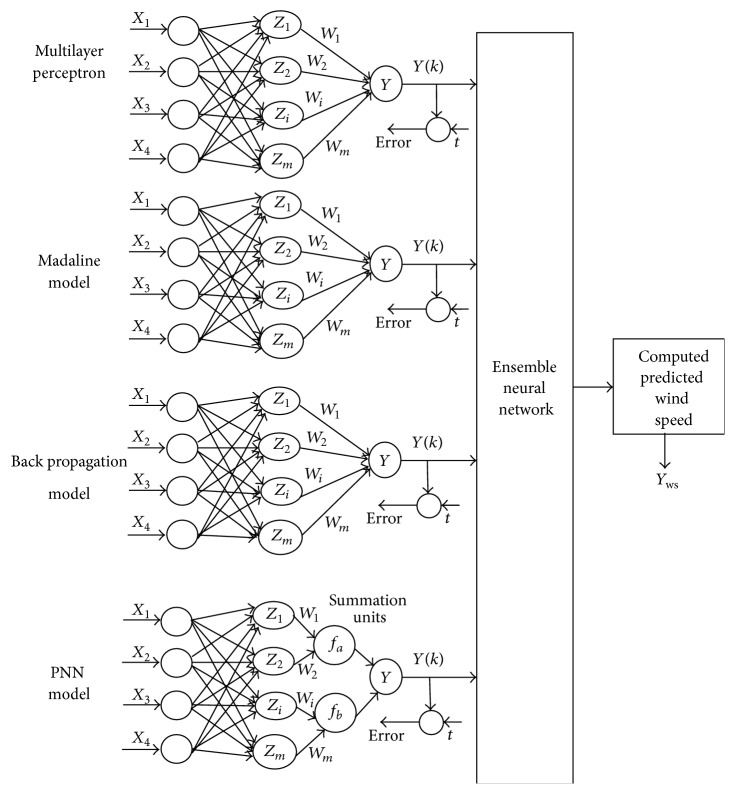
The proposed architectural model of ensemble neural network.

In artificial neural network modeling, ensemble neural networks are the ones that combine the outputs of the independently trained neural networks. In the proposed ensemble modeling, the independent neural networks considered include multilayer perceptron, multilayer adaptive linear neuron, back propagation neural network, and probabilistic neural network. Each of these independent neural networks (MLP, Madaline, BPN, and PNN) is trained using their respective training algorithms along with the proposed criterion of selecting hidden neurons incorporated. The outputs of each of these independent neural networks are averaged to achieve the better output with respect to the problem under consideration. This proposed methodology of ensemble neural network results in increasing the stability and generalization ability of the individual neural networks avoiding the local optima problem and delayed convergence of the network. The design of the ensemble neural network is performed in a manner to fix the appropriate number of hidden neurons with minimal error and inducing better accuracy and faster convergence. The proposed architecture model of ensemble neural network is as shown in Figure 2.

From Figure 2, it can be noted that the individual neural network models perform the independent computations on the input data received and the computed results are sent to the subsequent layer, where suitable processing takes place and finally the network output is determined. The evolved criteria are employed for identifying the number of hidden neurons in neural network design and the proposed ensemble approach is adopted for wind speed prediction application.

The operational procedure of each of the proposed ensemble neural networks is as given below.

*Module 1*. The multilayer perceptrons (MLP) are used with the supervised learning approaches and there exists a nonlinear activation function. The widely used nonlinear activation function in MLP is the logistic sigmoidal function. The MLP network also has various layers of hidden neurons and this hidden layer neuron is fixed employing the proposed criterion. The hidden neurons enable the ensemble MLP network to activate for highly complex tasks like wind speed forecasting. Each of the MLP layers is connected within them by synaptic weights.

*Module 2*. Single Adalines combine together, so that the output from certain Adalines becomes the input for few others; then the net becomes multilayer adaptive linear neuron. In ensemble Madaline processing, first the initialization of the weights between the input and the hidden units is done (these are small positive random by values). Then the considered input is presented, and based on the weighted interconnections the net input is computed for both the hidden units. Activation is then applied to obtain the outputs of the hidden units. The hidden output acts as input for the output layer and with suitable weighted interconnections, the net input and output of the output layer are computed. Then this is compared with that of the target and suitable weight updates are performed.

*Module 3*. In back propagation neural network module as shown in Figure 2, the input layer is connected to the hidden layer and the hidden layer is connected to the output layer by means of interconnected weights. Here during the back propagation phase of training, the signals are sent in the reverse direction. The increase in the number of hidden layers results in the computational complexity of the network and hence basically one hidden layer is used. The proposed criterion is incorporated into the training algorithm to fix the number of hidden neurons in the single hidden layer. In ensemble BPN processing, the bias is provided for both the hidden and the output layer, to act upon the net input to be calculated.

*Module 4*. The ensemble probabilistic neural net works on the concept of Bayesian classification and the estimation of probability density functions. Here the input vectors are classified into one of the two classes in a Bayesian optimal manner. In Figure 2, it can be noted that *f*_*A*_(*x*) serves as an estimator as long as the parent density is smooth and continuous. *f*_*A*_(*x*) approaches the parent density function as the number of data points used for the estimation increases. It should be noted that the function *f*_*A*_(*x*) represents the sum of Gaussian distributions. In this paper attempt is made to fix the number of pattern (hidden) units during the training process of the algorithm.

All the proposed four modules that comprise the ensemble model initiate its training process by learning from the normalized data. The training process is carried out until the error (performance metric) reaches a negligible value. The average value of MLP, Madaline, BPN, and PNN corresponding to the minimal error gives the final output of the proposed ensemble neural network.

### 4.2. The Proposed Training Algorithm of Ensemble Neural Network

The proposed training algorithm of ensemble neural network is as given below.

**Step 1 .**

Initialize the necessary parameters of the individual MLP, Madaline, BPN, and PNN.

**Step 2 .**

Introduce the proposed criterion to fix the number of hidden neurons into each of the training algorithms of individual neural network models.

**Step 3 .**

Present the input and target vector pair to the individual ensemble neural network models. The input-target vector pair corresponds to training datasets when training process is initiated and for testing the trained network, testing datasets are employed.

**Step 4 .**

Compute the net input of the individual nets and obtain their corresponding outputs by applying activation over the calculated net input. The outputs computed for each of the individual nets are given by *Y*_MLP_, *Y*_Madaline_, *Y*_BPN_, and *Y*_PNN_ and the individual nets are denoted as *H*_MLP_, *H*_Madaline_, *H*_BPN_, and *H*_PNN_.

**Step 5 .**

Develop the ensemble neural network as the aggregation of the individual neural network models and determine the final forecasted wind speed:

(2) Hensemblen=HMLPn,HMadalinen,HBPNn,HPNNn,Nensemblews=∑i=1nYnet_modelin,where  “n”—no.  of  individual  neural  nets,Nensemblews=YMLP+YMadaline+YBPN+YPNN4.

**Step 6 .**

Train each of the individual ensemble nets and compute the error value

(3) MSEensemble=∑i=1nMSEnet_modeln,MSEensemble=MSEMLP+MSEMadaline+MSEBPN+MSEPNN4.

**Step 7 .**

Select the appropriate hidden neurons to be placed in the hidden layer of each of the ensembles based on the minimum error performance.

**Step 8 .**

Output the selected criterion and the minimum MSE value.

**Step 9 .**

Test for stopping condition (the stopping condition can be the reaching point of minimum MSE or specified number of epochs).

The proposed ensemble neural network model is employed in this paper for forecasting wind speed and is presented in the following sections.

## 5. Numerical Experimentation and Simulation Results

The proposed methodology of ensemble neural networks is applied in this section for predicting wind speed. Ensemble model of neural networks involves the process of modeling the network architecture, training and testing process. The proposed modeling of ensemble neural network focuses on fixing the number of hidden neurons and thereby reducing the mean square error and predicting the accurate wind speed. Fundamentally, fixing the number of hidden neurons and thereby carrying out the prediction process enable the neural network to attain faster convergence with higher accuracy and reliability. Thus this work proposed 102 possible criteria to select the number of hidden neurons based on the computed error value during the learning process. Also, the wind speed is forecasted from the output of the ensemble neural network model after carrying out the denormalization procedure.

### 5.1. Description of Real Time Wind Farm Datasets

The real time wind farm data are obtained from Suzlon Pvt. Ltd., Coimbatore, Tamil Nadu, India, for a period from April 2013 to March 2015 pertaining to wind mills located in Palladam area. The height of the wind farm in Palladam area is 50 m. The proposed ensemble based wind speed prediction model has the essential inputs to be temperature, wind direction, relative humidity, and wind speed. The output of the proposed model is the predicted wind speed. 90,000 data samples are employed to develop the proposed model. In the total sample, 63,000 samples are employed for training process and 27,000 data samples are employed for testing process.  Table 1 presents the details of the wind input parameters. These input parameters act as the input neurons to be placed in the input layer of the proposed ensemble neural network model.  Table 2 shows the sample real time input wind farm data collected at the Palladam site. In Table 2, the direction of the wind is measured using a wind vane attached to a pole from north degree. The wind vane points the direction from which the wind blows. The direction of the wind is measured clockwise from true north and is represented in degrees. The wind speed is noted employing an anemometer. The temperature is basically observed using a thermometer. Hence, in this paper for forecasting wind speed, the essential inputs are considered to be wind direction, temperature, humidity, and wind speed.

### 5.2. The Proposed Wind Speed Prediction Ensemble NN Simulation

The ensemble neural network model is developed and simulated for the considered real time datasets. Each of the individual ensembles is trained until they reach the minimum error value to achieve better accuracy. The input variables and output variable that initiate the training process as input neurons and output neurons, respectively, of the ensemble models are as presented in Table 3.

To design the wind speed prediction model using the proposed ensemble neuronal model, the first step is to normalize the raw wind farm data. The process of normalization transforms the actual input data into normalized wind data. Fundamentally, normalization or scaling is carried out for improving the accuracy of numeric computation and resulting in a better accuracy rate. The wind farm data normalization is carried out using the min–max normalization approach [42]. For all the input parameters employed, their maximum and minimum values are noted and (4) is employed to perform the respective normalization operation:

(4) Xnorm=Xactual−Xmin_inputXmax_input−Xmin_inputXmax_target−Xmin_target+Xmin_target,

where *X*_actual_, *X*_min_input_, and *X*_max_input_ represent the actual input value and minimum and maximum input data, respectively. Also, *X*_max_target_ and *X*_min_target_ give the maximum and minimum target value of the wind farm data.

To design the ensemble neural network model, the necessary design parameters of each of the ensembles are to be set before initiating the training process.  Table 4 gives the design parameters initialized for the proposed ensemble neural network. The proposed ensemble NN model defines its architecture including the number of neurons to be placed in each layer and also the type of interconnections. Proper selection of parameters plays a vital role in avoiding the local minima problem during the training process. In the designed ensemble model, the input signals are processed and the net inputs of individual ensembles are calculated. The net input is the weighted sum of inputs and the respective activation function of the ensembles is employed to calculate the output of the network. Based on the computed output and desired set output, the error value is computed and based on the computed error, the weights pertaining to the ensembles are updated and the learning process is continued until the set stopping condition (minimum error value) is attained.

This paper focuses on performing accurate wind prediction for the real time wind farm data employing the proposed ensemble NN model. The individual ensembles are employed with each of the proposed criteria for fixing the number of hidden neurons. This paper proposes 102 criteria for the fixation of hidden neurons in the proposed individual ensemble neural network model.  Table 5 shows the proposed criteria developed to fix the number of hidden neurons in the designed ensemble network. The criteria are selected on satisfying the convergence theorem as presented in Appendix. Each of the criteria is applied to the individual ensembles. The mean square error is calculated for the individual ensembles. The average of the individual MSE of the ensembles gives the cumulative MSE value. The number of hidden neurons to be fixed is based on the criterion that possesses the lowest mean square error. It was not specific to choose 102 criteria; based on the analysis done several candidate criteria were selected and the criterion satisfying the convergence theorem with minimal MSE compared to that of the other candidate criteria was considered and presented in Table 5.

From the real time dataset, the training data is employed to forecast the wind speed and the testing data is used to evaluate the performance of the proposed model. Considering the 90,000 wind farm data samples, 70% of them, that is, 63,000 data samples, are employed for training process and the remaining 30% (27,000) are employed for testing the proposed model and predicting the wind speed. The proposed 102 criteria are applied to the proposed ensemble NN model one by one and MSE is evaluated. The criterion with the minimal MSE is selected as the candidate criterion to fix number of hidden neurons in the proposed ensemble NN model. The training dataset performs in ensemble neural network learning process and testing dataset performs in estimating the error.

From Table 5, it can be inferred that, for the candidate criteria (6*n* + 7)/(*n* − 3), the mean square error value is computed as 0.015150 employing the proposed ensemble neural network model. Appendix presents the proof for the chosen candidate criteria that achieved minimum MSE using the proposed ensemble NN model. Hence, the proposed ensemble model improves the accuracy and effectiveness for wind speed prediction application.  Table 6 presents a set of sample wind speed outputs computed during the testing process of the proposed ensemble NN model. From Table 6, it is observed that the actual output and the predicted output of the proposed model are approximately the same.

## 6. Discussion on the Simulated Results

**Figure 3:**
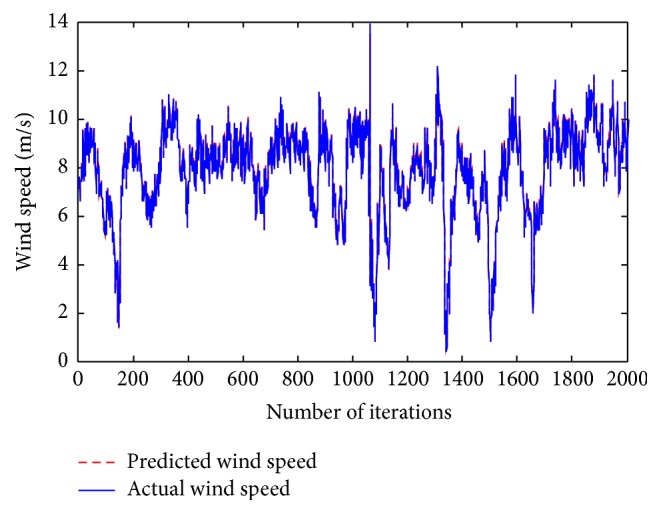
Comparison between the predicted and actual wind speed employing the proposed ensemble model.

The previous section presented the simulation results obtained using the proposed ensemble neural network model. The entire proposed neural network model is run in MATLAB R2009 environment and executed in Intel Core 2 Duo Processor with 2.27 GHz speed and 2.00 GB RAM. From Tables 6 and 7, it is clearly inferred that the proposed methodology achieves minimal MSE and the forecasted wind speed is noted to be in equivalence with that of the predicted wind speed.  Figure 3 shows the comparison made between the actual and predicted wind speed over the number of iterations. From Figure 3, it is noted that over 2000 iterations using the proposed methodology the actual and predicted wind speed are noted to be in accordance with each other.

**Figure 4:**
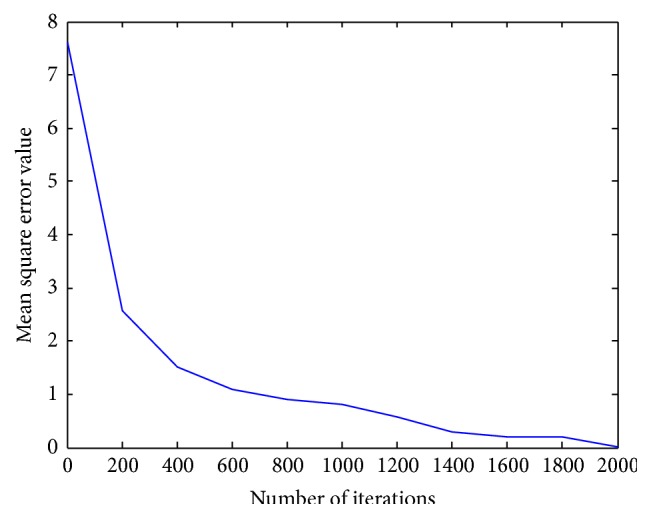
Computed MSE value using the proposed ensemble NN model.

Figure 4 presents the variation in the mean square error value with respect to the number of iterations using the proposed ensemble neural network model. From Figure 4, it can be noted that the MSE reaches a minimum of 0.01515 for the proposed ensemble model with the chosen hidden neuron criterion. Earlier approaches from the literature employed trial and error rule for computing the number of hidden neurons to be placed in the neural network. This proposed methodology worked on the mathematical foundation of convergence theorem and all the said criteria in Table 5 are noted to obey the convergence theorem. The selected criterion for fixing the number of hidden neurons is (6*n* + 7)/(*n* − 3), which employs 31 hidden neurons into the proposed ensemble model and achieves the better predicted wind speed with a minimal MSE of 0.015150.  Table 7 presents the comparison made between the proposed methodology and that of the existing approaches from the literature. It can be noted, from Table 7, that the proposed ensemble approach results in minimal mean square error in comparison with that of the earlier proposed approaches available in the literature. This proves the effectiveness of the proposed approach. Further, the simulation results from Table 6 prove that the predicted wind speed is in best agreement with that of the actual wind speed values.

## 7. Conclusion

A novel ensemble neural network model comprised of individual ensembles is developed in this paper for wind speed prediction application. The individual ensembles modeled include multilayer perceptron, Madaline, back propagation neural network, and probabilistic neural network. Each of the ensembles is trained employing the proposed selection criterion for fixing hidden neurons and the average of the individual ensembles resulted in the development of the proposed ensemble neural network model. From the simulation results, it is well noted that the proposed ensemble neural network model resulted in minimal mean square error value proving its effectiveness over the earlier existing approaches from the literature. The proposed approach aimed at implementing the fixation of proper number of hidden neurons in ensemble neural network for wind speed prediction in renewable energy systems.

## Figures and Tables

**Table 1:** Details of the wind input parameters for the proposed model.

Sl. number	Input parameters	Unit	Range of the parameter
1	Temperature	Degree Celsius	24–36
2	Wind direction	Degree	1–350
3	Wind speed	m/s	1–16
4	Relative humidity	Percentage	52–90

**Table 2:** Real time wind farm data samples (Suzlon Pvt. Ltd.).

Temperature (degree Celsius)	Wind direction (degree)	Wind speed (m/s)	Relative humidity (%)
27.6	109.7	5.1	63.67
27.6	108.3	5.1	63.12
27.6	111.1	5.5	65.91
26.8	109.7	4.9	65.00
26.8	109.7	4.9	65.48
26.8	112.5	5.2	66.78
26.8	115.3	5	64.31
26.8	112.5	4.6	63.01
26.8	108.3	4.4	62.59
26.7	111.1	3.9	62.44
26.7	119.5	3.8	62.02
26.7	113.9	4	65.79
26.7	113.9	4	65.72
26.7	108.3	4.1	66.03
26.7	90	3.8	64.50
25.9	78.8	3.3	63.98
25.9	78.8	3.6	64.76
25.9	83	4.6	65.33
25.9	80.2	3.8	65.10
25.9	81.6	2.7	60.25

**Table 3:** Input and output variables of the proposed ensemble neural model.

Input variable	Parameter description	Output variable	Parameter description
*X*_1_	Temperature	*Y*_ws_	Predicted wind speed
*X*_2_	Wind direction		
*X*_3_	Wind speed		
*X*_4_	Relative humidity		

**Table 4:** Design parameters of the proposed ensemble NN model.

Sl. number	Proposed individual ensemble NN model	Design parameters	Set values of design parameters
1	Multilayer perceptron (MLP) network	Inputs	4
		Number of iterations	2000
		Learning rate	0.2
		Threshold	1
		Activation function	Binary linear activation function

2	Madaline model	Inputs	4
		Number of iterations	2000
		Learning rate	0.2
		Number of hidden layer	1

3	Back propagation neural network (BPN)	Inputs	4
		Number of iterations	2000
		Learning rate	0.3
		Momentum factor	0.7
		Activation function	Binary sigmoidal function

4	Probabilistic neural network (PNN)	Inputs	4
		Number of iterations	2000
		Smoothing factor	6.1

**Table 5:** Proposed criteria with computed mean square error for fixing the hidden neurons in ensemble neural network model.

Proposed criteria for fixing number of hidden neurons	Number of hidden neurons	Mean square error				
		MLP	Madaline	BPN	PNN	Ensemble NN
(3(*n* ^2^ + 7) + 5)/(*n* ^2^ − 15)	74	0.121	0.013	0.1573	0.025	0.079075
3*n*/(*n* − 1)	4	0.059	0.082	0.0547	0.067	0.065675
(4*n* + 1)/(*n* − 3)	17	0.578	0.021	0.8859	0.038	0.380725
(5(*n* ^2^ + 1) + 5)/(*n* ^2^ − 15)	90	1.3	0.874	0.2731	0.211	0.664525
(2*n* + 5)/(*n* − 3)	13	0.009	0.188	0.1527	0.055	0.101175
(4(*n* ^2^ + 1) + 1)/(*n* ^2^ − 15)	69	0.013	0.19	2.0259	0.091	0.579975
(8*n* − 3)/(*n* − 3)	29	0.1	0.03	6.7517	0.089	1.742675
(3(*n* ^2^ + 1) + 1)/(*n* ^2^ − 15)	52	0.007	0.82	8.6577	0.0278	2.378125
6*n*/(*n* − 2)	12	0.2214	0.556	2.0249	0.015	0.704325
(3(*n* ^2^ + 3) + 2)/(*n* ^2^ − 15)	59	0.4621	0.077	3.68*E* − 04	0.0011	0.135142
*n*/(*n* + 1)	1	0.0995	0.07	3.00*E* − 05	2.13*E* − 04	0.042435
(4*n* ^2^ + 4)/(*n* ^2^ − 15)	80	0.1363	0.53	0.0077	0.1071	0.195275
(9*n* + 5)/(*n* − 3)	41	0.2578	0.449	2.98*E* − 05	0.025	0.182957
(4(*n* ^2^ + 3) − 1)/(*n* ^2^ − 15)	75	0.1934	0.781	8.53*E* − 06	4.06*E* − 04	0.243703
(3(*n* ^2^ + 1) + 3)/(*n* ^2^ − 15)	54	0.772	0.81	0.0222	5.08*E* − 04	0.401177
(3*n* + 4)/(*n* − 3)	16	0.188	0.026	0.0014	2.31*E* − 04	0.053907
(5(*n* ^2^ + 1) + 3)/(*n* ^2^ − 15)	88	1.418	0.578	1.25*E* − 06	2.60*E* − 04	0.499065
(9*n* + 6)/(*n* − 2)	21	0.08	0.53	0.4513	0.0735	0.283700
(4*n* ^2^ + 8)/(*n* ^2^ − 8)	9	0.188	1.024	0.0112	0.0771	0.325075
4*n*/(*n* − 2)	8	0.135	0.0113	0.0094	0.0422	0.049475
(8*n* + 1)/(*n* − 3)	33	0.29	0.0084	2.19*E* − 04	0.0366	0.083804
(5(*n* ^2^ + 4) + 1)/(*n* ^2^ − 15)	101	0.071	0.0135	3.79*E* − 05	0.1188	0.050834
(5(*n* ^2^ + 2) + 5)/(*n* ^2^ − 15)	95	0.29	0.008	2.70*E* − 05	0.2088	0.126706
(4(*n* ^2^ + 5) + 1)/(*n* ^2^ − 15)	85	0.091	0.0848	4.05*E* − 04	0.001	0.044301
(11*n* + 6)/(*n* − 3)	50	1.1039	0.0727	0.0697	0.1	0.336575
(5(*n* ^2^ + 1) + 7)/(*n* ^2^ − 15)	92	0.416	0.0695	0.116	0.305	0.226625
(5(*n* ^2^ + 1) + 3)/(*n* ^2^ − 8)	11	0.5746	0.0907	0.088	0.05	0.200825
(8*n* + 3)/(*n* − 3)	35	0.1611	0.0758	0.0497	0.0933	0.094975
(3*n* ^2^ + 8)/(*n* ^2^ − 8)	7	0.4042	0.0457	4.08*E* − 06	0.5498	0.249926
(4(*n* ^2^ + 4) + 4)/(*n* ^2^ − 15)	84	0.5825	0.0547	8.86*E* − 05	0.2613	0.224647
(9*n* + 2)/(*n* − 3)	38	0.4303	0.0232	0.0231	0.578	0.263650
(6*n* ^2^ + 1)/(*n* ^2^ − 15)	97	0.7184	0.0155	6.25*E* − 08	0.09	0.205975
(7*n* + 2)/(*n* − 3)	30	0.3417	0.0171	1.25*E* − 06	0.084	0.110700
(11*n* + 2)/(*n* − 3)	46	0.467	0.049	4.82*E* − 06	0.136	0.163001
(6*n* + 3)/(*n* − 3)	27	0.151	0.0723	2.70*E* − 08	0.2279	0.112800
(3(*n* ^2^ + 7) + 1)/(*n* ^2^ − 15)	70	0.2621	0.0278	3.23*E* − 06	0.6392	0.232275
(8*n* + 8)/(*n* − 2)	20	0.3376	0.035	4.48*E* − 05	0.3536	0.181561
(5(*n* ^2^ + 2) + 1)/(*n* ^2^ − 15)	91	0.1544	0.0083	0.5036	0.661	0.331825
11*n*/(*n* − 3)	44	0.195	0.0161	0.0095	1.63	0.462650
(4(*n* ^2^ + 3) + 1)/(*n* ^2^ − 15)	77	0.2874	0.0118	0.0049	1.418	0.430525
(7*n* − 2)/(*n* − 3)	26	0.02	0.0092	0.0043	0.881	0.228625
(3(*n* ^2^ + 3) + 3)/(*n* ^2^ − 15)	60	0.32	0.0113	4.73*E* − 04	0.0414	0.093293
(3(*n* ^2^ + 5) + 3)/(*n* ^2^ − 15)	66	0.539	0.0084	5.56*E* − 04	0.0285	0.144114
(5(*n* ^2^ + 3) − 1)/(*n* ^2^ − 15)	94	0.74	0.0135	3.88*E* − 04	0.0541	0.201997
2.5*n*/(*n* + 1)	2	0.04	0.0758	3.75*E* − 06	0.0651	0.045225
(11*n* + 4)/(*n* − 3)	48	0.128	0.0881	4.90*E* − 07	0.0959	0.078000
(4*n* ^2^ + 3)/(*n* ^2^ − 15)	67	0.171	0.1349	3.82*E* − 06	0.0798	0.096425
(4*n* ^2^ + 1)/(*n* ^2^ − 15)	65	0.83	0.1079	0.5498	0.0487	0.384100
(3*n* ^2^ + 5)/(*n* ^2^ − 15)	53	0.171	0.1342	0.2613	0.0084	0.143725
(4(*n* ^2^ + 2) + 1)/(*n* ^2^ − 15)	73	0.0907	0.2272	0.2808	0.0135	0.153050
(4(*n* ^2^ + 5) + 3)/(*n* ^2^ − 15)	87	0.0758	0.0879	0.772	0.008	0.235925
(7*n* + 6)/(*n* − 3)	34	0.0881	0.0044	0.188	0.0116	0.073025
(5(*n* ^2^ + 2) − 1)/(*n* ^2^ − 15)	89	0.1349	2.5344	1.418	0.086	1.043325
(3(*n* ^2^ + 2) + 4)/(*n* ^2^ − 15)	58	0.1079	0.0029	0.08	0.0907	0.070375
(6*n* ^2^ + 6)/(*n* ^2^ − 15)	102	0.1342	3.73*E* − 06	0.188	0.0758	0.099500
(3(*n* ^2^ + 4) + 1)/(*n* ^2^ − 15)	61	0.0106	1.15*E* − 06	0.0314	0.0586	0.025150
9*n*/(*n* − 2)	18	0.0113	2.69*E* − 08	0.2895	3.73*E* − 06	0.075200
(3*n* ^2^ + 7)/(*n* ^2^ − 15)	55	0.0084	0.0055	0.1354	1.15*E* − 06	0.037325
(6*n* + 7)/(*n* − 3)	**31**	**0.0135**	**0.0259**	**0.01**	**0.0112**	**0.015150**
(4*n* + 12)/(*n* − 2)	14	0.0446	2.20*E* − 04	0.056	0.0075	0.027080
(3(*n* ^2^ + 3) + 5)/(*n* ^2^ − 15)	62	0.1238	0.0072	0.578	2.209	0.729500
(10*n* + 5)/(*n* − 3)	45	0.1812	0.0178	0.541	0.0019	0.185475
2*n*/(*n* − 1)	3	0.0359	1.8132	0.136	1.012	0.749275
(9*n* + 7)/(*n* − 3)	43	0.0618	5.70*E* − 04	0.01	0.0244	0.024192
(6*n* ^2^ + 3)/(*n* ^2^ − 15)	99	0.217	0.0222	2.199	6.05*E* − 04	0.609701
(3(*n* ^2^ + 2) + 3)/(*n* ^2^ − 15)	57	0.0457	0.0014	1.418	0.0418	0.376725
(3*n* ^2^ + 1)/(*n* ^2^ − 15)	49	0.0547	1.25*E* − 06	0.01	0.1089	0.043400
(5(*n* ^2^ + 3) + 1)/(*n* ^2^ − 15)	96	0.082	0.5036	0.102	0.6826	0.342550
(*n* + 6)/(*n* − 2)	5	0.0623	0.0095	0.281	0.5188	0.217900
(3(*n* ^2^ + 5) + 5)/(*n* ^2^ − 15)	68	0.1264	0.0049	0.419	0.4704	0.255175
(5*n* ^2^ + 1)/(*n* ^2^ − 15)	81	0.1149	0.0043	0.071	3.9044	1.023650
(6*n* + 1)/(*n* − 3)	25	0.049	0.098	0.83	0.3826	0.339900
(3(*n* ^2^ + 2) + 2)/(*n* ^2^ − 15)	56	0.0723	3.10*E* − 05	0.135	0.736	0.235832
(10*n* + 7)/(*n* − 3)	47	0.0278	3.68*E* − 04	0.075	0.7182	0.205342
(4(*n* ^2^ + 2) + 4)/(*n* ^2^ − 15)	76	0.035	0.102	0.032	0.2251	0.098525
4.5*n*/(*n* − 1)	6	0.0125	0.0637	3.00*E* − 05	0.1006	0.044207
(5*n* + 4)/(*n* − 3)	24	0.0101	3.293	2.10*E* − 05	0.3915	0.923655
8*n*/(*n* − 3)	32	0.0083	0.0845	3.90*E* − 04	0.1573	0.062622
(4(*n* ^2^ + 3) + 3)/(*n* ^2^ − 15)	79	0.0161	3.2678	9.60*E* − 04	0.2291	0.878490
(8*n* + 5)/(*n* − 3)	37	0.0118	2.8695	1.82*E* − 04	1.7917	1.168295
(10*n* + 2)/(*n* − 3)	42	0.5746	1.9449	2.68*E* − 04	0.0737	0.648367
(4(*n* ^2^ + 4) + 2)/(*n* ^2^ − 15)	82	0.1611	1.7361	4.84*E* − 04	0.0987	0.499096
(8*n* + 7)/(*n* − 3)	39	0.4042	0.6691	4.05*E* − 04	0.5597	0.408351
(8*n* + 6)/(*n* − 2)	19	0.4438	0.0585	0.0034	0.0565	0.14055
(3*n* ^2^ + 3)/(*n* ^2^ − 15)	51	0.071	1.4727	4.73*E* − 04	2.5634	1.02689
(4*n* + 6)/(*n* − 3)	22	0.031	0.9435	9.26*E* − 04	0.0314	0.25170
(4(*n* ^2^ + 1) + 3)/(*n* ^2^ − 15)	71	0.075	0.1603	4.24*E* − 06	0.2895	0.13120
10*n*/(*n* − 3)	40	0.032	0.0799	4.96*E* − 07	0.1354	0.06182
(5(*n* ^2^ + 2) + 3)/(*n* ^2^ − 15)	93	0.071	0.2478	1.26*E* − 07	0.2613	0.14502
(4(*n* ^2^ + 4) − 2)/(*n* ^2^ − 15)	78	0.29	0.2647	0.5036	0.2808	0.33477
(5(*n* ^2^ + 3) + 5)/(*n* ^2^ − 15)	100	0.091	0.2325	0.0095	0.276	0.15225
5*n*/(*n* − 2)	10	1.024	0.221	0.0049	0.5299	0.44495
(7*n* + 2)/(*n* − 2)	15	0.136	0.009	0.9315	0.0752	0.28792
(3(*n* ^2^ + 7) + 3)/(*n* ^2^ − 15)	72	1.01	0.088	0.2581	0.0445	0.35015
(5*n* ^2^ + 3)/(*n* ^2^ − 15)	83	2.199	1.0171	0.3852	0.1892	0.94762
9*n*/(*n* − 3)	36	1.418	0.2045	0.2573	0.1709	0.51267
(3(*n* ^2^ + 5) + 1)/(*n* ^2^ − 15)	64	0.032	0.33	1.0982	0.076	0.38405
(5(*n* ^2^ + 1) + 1)/(*n* ^2^ − 15)	86	0.82	0.8262	0.463	0.0397	0.53722
(3(*n* ^2^ + 4) + 3)/(*n* ^2^ − 15)	63	0.228	0.7203	0.33	0.005	0.32082
(4*n* + 7)/(*n* − 3)	23	0.135	0.59	0.8262	0.0028	0.38850
7*n*/(*n* − 3)	28	0.195	7.665	0.951	0.0013	2.20307
(5(*n* ^2^ + 3) + 3)/(*n* ^2^ − 15)	98	0.2874	0.327	0.031	0.253	0.22460

**Table 6:** Actual and predicted output of the proposed ensemble NN model.

Actual output	Predicted output	Actual output	Predicted output	Actual output	Predicted output	Actual output	Predicted output
2.7891	2.8	3.7970	3.8	2.8566	2.9	1.2569	1.3
2.7900	2.8	3.5600	3.6	2.7809	2.8	1.4003	1.4
2.9832	3	3.3009	3.3	2.1897	2.2	0.6021	0.5
3.1187	3.2	3.5985	3.6	0.9632	0.7	1.5980	1.6
3.0657	3.1	2.6894	2.7	2.3196	2.4	2.6652	2.7
2.9003	2.9	3.0321	3.1	1.2109	1.2	1.5167	1.5
2.6754	2.7	3.0987	3.1	2.4760	2.5	2.5590	2.6
1.3125	1.3	3.1786	3.2	1.9974	2	2.2980	2.3
2.1876	2.2	3.3958	3.4	1.8876	1.9	1.7657	1.8
2.6592	2.7	4.2176	4.2	0.3877	0.4	2.1900	2.2
2.5782	2.6	3.5998	3.6	1.5943	1.6	3.4788	3.5
2.7931	2.8	3.8063	3.8	0.6122	0.4	2.0098	2.1
3.1788	3.2	3.5811	3.6	0.5988	0.6	4.3981	4.4
3.5783	3.6	2.8091	2.9	0.6690	0.7	3.8975	3.9
3.5900	3.6	2.4125	2.5	0.5413	0.4	2.8831	2.9
3.8023	3.8	3.1977	3.2	1.5984	1.6	2.9980	3
4.0001	4	3.2699	3.3	0.5922	0.6	2.5926	2.6
4.0955	4.1	2.9987	3	0.6087	0.6	0.3921	0.4
4.3756	4.4	3.1079	3.1	0.4660	0.4	0.6547	0.5
4.2056	4.2	2.3822	2.4	1.0053	0.9	0.4238	0.4

**Table 7:** Comparison of MSE for the approaches in the existing and proposed ensemble NN model.

S. number	Various approaches	Criteria employed for fixing number of hidden neurons	MSE
1	Li et al. method [44]	$N_{h}=\left[\left(\surd 1+8n\right)-\frac{1}{2}\right]$	0.1532
2	Tamura and Tateishi method [45]	*N*_*h*_ = *N* − 1	0.2179
3	Fujita method [46]	$N_{h}=\frac{K\log\left(\|P_{c}Z\|/C\right)}{\log s}$	0.1982
4	Zhang et al. method [47]	$N_{h}=\frac{2^{n}}{n+1}$	0.2246
5	Ke and Liu method [48]	$N_{h}=\frac{N_{in}+\surd N_{p}}{L}$	0.0691
6	Xu and Chen method [49]	$N_{h}=C_{f}{\left(\frac{N}{d}\log N\right)}^{1/2}$	0.0731
7	Shibata and Ikeda method [50]	*N*_*h*_ = √*N*_*i*_ *N*_*o*_	0.1076
8	Hunter et al. method [51]	*N*_*h*_ = 2^*n*^ − 1	0.1627
9	Sheela and Deepa method [52]	$N_{h}=\frac{4n^{2}+3}{n^{2}-8}$	0.0587
10	*Proposed ensemble NN model*	$\frac{6n+7}{n-3}$	**0.0151**

## Appendix

A considered sequence is said to be convergent [43] if it has a finite limit. When the sequence does not converge to any number then it is said to be divergent. A convergent sequence *Z*_1_, *Z*_2_,…, *Z*_*n*_ is one that has a limit *C*, written as lim_*n*→*α*_ *Z*_*n*_ = *C*. By definition of limit, this means that, for every *ε* > 0, it can find *N* such that

(A.1) $$\begin{array}{c} \left|\;Z_{n}-C\;\right|<\varepsilon \;\forall n>N. \end{array}$$

In the proposed approach, consider numerous criteria where the number of input neurons is represented as “*n*.” All criteria are satisfied with that of the convergence theorem. Basically, the convergence sequence has a finite limit and the other sequence is called a divergent sequence.

For the considered various criteria, all criteria are satisfied with convergence theorem and hence are incorporated into the ensemble neural network model. The proof that the proposed criterion (6*n* + 7)/(*n* − 3) satisfies the convergence theorem is as follows.

The sequence is

(A.2) an=6n+7n−3,limn→∞6n+7n−3=6n1+7/6nn1−3/n=61+01−0=6,a  finite  value.

Henceforth, the terms of sequence represented by (6*n* + 7)/(*n* − 3) are bounded and the sequence has a limit value and the sequence is convergent.
